# Mitochondrial Oxidative Phosphorylation System Dysfunction in Schizophrenia

**DOI:** 10.3390/ijms26094415

**Published:** 2025-05-06

**Authors:** Constanza Morén, David Olivares-Berjaga, Albert Martínez-Pinteño, Miquel Bioque, Natàlia Rodríguez, Patricia Gassó, Lourdes Martorell, Eduard Parellada

**Affiliations:** 1 Barcelona Clínic Schizophrenia Unit (BCSU), Neuroscience Institute, Hospital Clínic of Barcelona, 08036 Barcelona, Spain; mbioque@clinic.cat (M.B.); eparella@clinic.cat (E.P.); 2 Schizophrenia Research Group, Institut d’Investigacions Biomèdiques August Pi i Sunyer (IDIBAPS), 08036 Barcelona, Spain; olivares99@ub.edu (D.O.-B.); albert.martinez@ub.edu (A.M.-P.); nrodriguezfe@ub.edu (N.R.); 3 Department of Fundamental and Clinical Nursing, Nursing Faculty, University of Barcelona, 08036 Barcelona, Spain; 4 Centro de Investigación Biomédica en Red de Salud Mental (CIBERSAM), Instituto de Salud Carlos III, 28029 Madrid, Spain; lourdes.martorell@urv.cat; 5 Basic and Clinical Practice Department, University of Barcelona, 08036 Barcelona, Spain; 6 Department of Medicine, University of Barcelona, 08036 Barcelona, Spain; 7 Hospital Universitari Institut Pere Mata (HUIPM), Institut d’Investigació Sanitària Pere Virgili (IISPV-CERCA), Universitat Rovira i Virgili (URV), 43206 Reus, Spain

**Keywords:** bioenergetics, metabolism, mitochondria, OXPHOS, schizophrenia

## Abstract

Schizophrenia (SCZ) is a severe, chronic mental disorder of unknown etiology and limited therapeutic options. Bioenergetic deficits in the oxidative phosphorylation system (OXPHOS) during early postnatal brain development may underlie disrupted neuronal metabolism and synaptic signaling, contributing to the neurodevelopmental and behavioral disturbances observed in patients. This narrative review summarizes updated evidence linking mitochondrial-OXPHOS dysfunction to SCZ pathophysiology. The novelty lies in the focus on OXPHOS dysfunction at the enzymatic/functional level, rather than on genetic, transcriptional, or oxidative parameters. While complex I impairment has long been highlighted and proposed as a peripheral marker of the disease, recent studies also report alterations in other OXPHOS complexes and their precursors. These findings suggest that OXPHOS dysfunction is not isolated to a single enzymatic component but affects broader mitochondrial function, alongside oxidative stress, contributing to disease progression through mechanisms involving apoptosis, accelerated aging, and synaptic deterioration. OXPHOS dysfunction in both central and peripheral tissues further supports its relevance to SCZ. Overall, the literature points to mitochondrial OXPHOS abnormalities as a significant biological feature of SCZ. Whether these alterations are causal factors or consequences of disease processes remains unclear. Understanding OXPHOS dysregulation may open new avenues for targeted therapies.

## 1. Introduction

**Figure 1:**
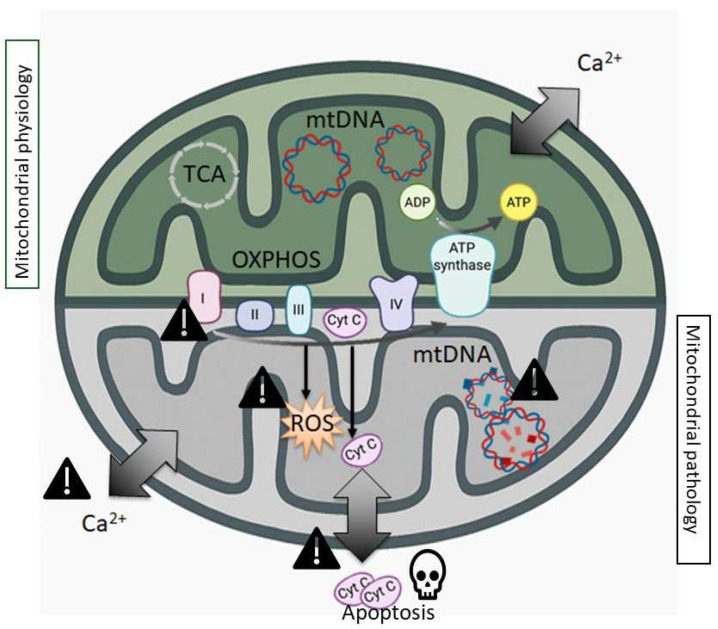
Upper half, mitochondrial physiology with electron transport chain and OXPHOS in the inner mitochondrial membrane, Krebs cycle providing molecules with reducing power to feed the mitochondrial respiratory chain, and intact mitochondrial genomes, located in the mitochondrial matrix. Bottom half, mitochondrial pathology with impaired mtDNA molecules, alterations in calcium homeostasis, cytochrome c release inducing apoptosis, and dysfunction of the OXPHOS generating ROS. The black warning symbols in the figure indicate the main points of dysfunction within the pathological context (at the bottom). ADP, adenosine diphosphate; ATP, adenosine triphosphate; Ca^2+^, calcium; CoQ, coenzyme Q; cyt C, cytochrome c; H^+^, hydrogen protons; H_2_O, water; mtDNA, mitochondrial DNA; NAD+, nicotinamide adenine dinucleotide; NADH, reduced form of nicotinamide adenine dinucleotide; O_2_, oxygen; OXPHOS: oxidative phosphorylation system; ROS: reactive oxygen species; TCA, tricarboxylic acid cycle.

The oxidative phosphorylation system (OXPHOS) is a fundamental biochemical process that occurs in the mitochondria of eukaryotic cells. Key components of OXPHOS include the electron transport chain (ETC)—consisting of protein complexes I (reduced form of nicotinamide adenine dinucleotide, NADH dehydrogenase), II (succinate dehydrogenase), III (cytochrome c reductase), and IV (cytochrome c oxidase)—and the proton gradient, adenosine triphosphate (ATP) synthase. Many proteins in the OXPHOS pathway are iron-sulfur proteins. OXPHOS involves the coupling of electron transport through the ETC with the synthesis of ATP, the primary energy currency of the cell [1]. Thus, OXPHOS orchestrates the conversion of metabolic substrates into cellular energy. This process involves the sequential transfer of electrons through the aforementioned protein complexes, embedded in the inner mitochondrial membrane, ultimately leading to the reduction of molecular oxygen to water at complex IV. Simultaneously, this electron flow creates a proton gradient across the membrane, establishing an electrochemical potential. ATP synthase then uses the energy stored in this proton gradient to catalyze the phosphorylation of adenosine diphosphate (ADP) to ATP [1]. As a result, OXPHOS is the primary mechanism for cellular energy production in aerobic organisms and is essential for a variety of life-sustaining physiological processes (Figure 1, left half).

Schizophrenia (SCZ) is a persistent and severe mental disorder characterized by disturbances in thought, perception, emotion, and behavior, often manifesting as hallucinations, delusions, disorganized thinking, and impaired social functioning [2,3]. During early postnatal brain development, deficits in bioenergetics may lay the groundwork for disrupted neuronal metabolism or synaptic signaling, potentially contributing to the increased incidence of developmental and behavioral deficits observed in affected individuals. A growing body of evidence suggests that mitochondrial dysfunction may be a central aspect of SCZ pathology [4,5]. In particular, alterations in complex I function have been identified as prominent features, leading to their consideration as peripheral biomarkers of the disease [6,7]. These mitochondrial changes may underlie clinical psychiatric manifestations, as recent studies have linked mitochondrial hyperactivity to social behavioral impairments [8]. Metabolic shift from glycolysis to mitochondrial OXPHOS, may be impaired in SCZ due to mitochondrial dysfunction, leading to altered neuronal energy homeostasis and synaptic deficits [9]. This state of stress-induced hypermetabolism has also been associated with accelerated aging [10]. Notably, a significant reduction in the NAD+/NADH ratio, measured using a novel in vivo 31P magnetic resonance spectroscopy (MRS) technique was found in both chronically ill and first-episode SCZ patients compared to healthy controls. These findings provide compelling evidence for a redox imbalance in the brain across all phases of SCZ [11]. OXPHOS dysfunction can generate reactive oxygen species (ROS), which can ultimately promote oxidative damage within various cellular structures [1], contributing to disease, aging, and senescence. Low levels of antioxidant systems, particularly glutathione, have been found in SCZ [12] (Figure 1, right half). On the other hand, parvalbumin-positive interneurons (which play an essential role in maintaining a finely tuned excitation–inhibition balance in the brain and are known to play a role in the pathophysiology of SCZ) are particularly susceptible to oxidative stress, and oxidative stress can also cause deficits in myelination [13,14]. In addition, oxidative stress has been linked to apoptotic mechanisms that underlie the accelerated dendritic pruning around the onset of SCZ [15].

SCZ has a heritability of 60–80%, with a significant proportion of this attributable to common risk alleles [16]. The variability in individual risk is largely influenced by genetic factors, including a wide range of common alleles [17], rare copy number variants (CNVs) [17], and rare coding variants (RCVs) [18,19,20]. This genetic predisposition interacts with environmental factors to contribute to the complex etiology and variable expression observed in SCZ spectrum disorders. In the largest GWAS study conducted in SCZ patients, genetic variants in the aspartate beta-hydroxylase domain containing 1 gene or *ASPHD1* (redox reactions), aspartyl-tRNA synthetase 2, mitochondrial gene or *DARS2* (mitochondrial enzyme involved in aspartyl tRNA aminoacylation), and ecto-NOX disulfide thiol exchanger 1 gene or *ENOX* (oxidase activity via NADH), among others, were associated with a higher risk to present the disease [16].

The strong influence of genetic factors on the penetrance of SCZ highlights its hereditary nature. This genetic predisposition involves not only nuclear DNA but also mitochondrial DNA, suggesting that mitochondrial function may play a role in the disease’s pathophysiology.

Regarding the nuclear genome, the congenital disorder 22q11.2 deletion syndrome (22qDS), characterized by a hemizygous deletion of 1.5–3 Mb on chromosome 22 at locus 11.2, is the most common microdeletion disorder (estimated prevalence of 1 in 4000) and one of the most important risk factors for SCZ. Alterations in nine of the ∼thirty genes involved in 22qDS have the potential to disrupt mitochondrial metabolism (*COMT*, *UFD1L*, *DGCR8*, *MRPL40*, *PRODH*, *SLC25A1*, *TXNRD2*, *T10*, and *ZDHHC8*) [21]. Mitochondrial deficits have been observed in human iPSC-derived neurons from patients with 22q11.2 deletion syndrome and SCZ [22]. Deficits in bioenergetics during early postnatal brain development may disrupt neuronal metabolism or synaptic signaling [9]. The 1.6 megabase deletion on chromosome 3q29 (3q29Del) is a strongly identified genetic risk factor for SCZ, although the effects of this variant on neurodevelopment are not well understood. Systematic pathway analysis has implicated dysregulation of mitochondrial function and energy metabolism with a lack of metabolic flexibility (toward glycolysis at the expense of oxidative metabolism via OXPHOS) in human and animal models [23]. The examples presented here (22q11.2 and 3q29) demonstrate nuclear genomic alterations as a relevant risk factor for disease development, and in each case, mitochondrial involvement is evident [9,23].

Moving from the nuclear genome to the mitochondrial genome (mtDNA), mitochondrial variants have also been associated with SCZ [24]. On the one hand, evidence of maternal inheritance and the presence of SCZ symptoms in patients with a mitochondrial disorder associated with a mtDNA mutation suggests that mtDNA is involved in SCZ [25]. On the other hand, the association of specific variants has been reported at the molecular level. In particular, the mtDNA 10398 A/G polymorphism is known to affect the regulation of mitochondrial calcium levels involved in energy production, and is associated with psychiatric disorders [26]. The literature reports the case of a patient harboring the m.A3243G mtDNA mutation with clinical and magnetic resonance imaging findings of Wernicke–Korsakoff syndrome who developed SCZ with predominantly negative symptoms some years later [27].

In addition to the genetic framework, several other biological factors may regulate OXPHOS and be associated with the pathogenesis of SCZ. These include hormonal factors, oxidative damage driven by antioxidant defenses, and calcium homeostasis: (I) Hormonal regulation of OXPHOS occurs in the brain and may underlie the basis of neuropathology [28]. The involvement of hormones in the pathogenesis of SCZ has long been suspected because the psychosis differs in females and males and the disease usually occurs shortly after puberty [29,30]. (II) The suboptimal function of OXPHOS, together with alterations in cellular antioxidant defenses such as glutathione (GSH) deficits, may lead to oxidative stress, which in turn has also been widely implicated in the pathogenesis of SCZ [12,13], to the extent that it is considered a potent target of current psychopharmacotherapy [31]. Lipid peroxidation disorders have been observed in SCZ patients with persistent paranoid symptoms who are refractory to treatment and one year after the first psychotic episode [32,33] as well as in other subtypes of affected patients [34]. Indices of endogenous oxidative and antioxidative processes measured in the plasma of SCZ patients suggest a possible role for increased oxidative stress and decreased enzymatic antioxidants, as potential alterations in the core pathophysiology of SCZ [35]. (III) In the context of calcium homeostasis, S100B is a glial-secreted calcium-binding protein produced primarily by astrocytes in the nervous system. This protein has been implicated in the calcium-dependent regulation of a variety of intracellular functions, including protein phosphorylation, enzyme activities, cell proliferation and differentiation, cytoskeletal dynamics, structural organization of membranes, intracellular calcium homeostasis (a function shared with mitochondria), inflammation, and protection against oxidative cell damage. Elevated levels of S100B levels in blood or cerebrospinal fluid have been observed in patients with SCZ [36]. In addition, mitochondrial calcium levels are involved in apoptotic pathways and neuronal signaling [37,38].

The idea that the OXPHOS system may play a critical role in the etiology of SCZ has also led to studies of mitotherapy aimed at this target [39]. Mitotherapy has shown promising results in experimental models of SCZ. Isolated mitochondrial transfer improved neuronal differentiation of SCZ-derived induced pluripotent stem cells and rescued deficits in a rat model of the disease [40]. Thus, mitochondrial transplantation may eventually be useful as a therapeutic approach for SCZ, correcting brain pathology and restoring cognitive deficits associated with the disease. In this context, our main objective was to perform a narrative review summarizing the most important alterations identified at the level of OXPHOS in clinically diagnosed chronic SCZ.

## 2. Literature Selection and Scope

This is a narrative review compiling studies on OXPHOS alterations in the context of SCZ. Articles were identified by searching for titles in the Web of Science (WoS), PUBMED (MEDLINE), and SCOPUS databases by using the following keywords and Boolean operators: “schizophrenia” AND “OXPHOS” OR “oxidative phosphorylation system”. We avoided psychosis, first-episode psychosis, or other related terms because our interest was mainly focused on those chronic cases with established clinically diagnosed SCZ, thus excluding cases with a first episode that might eventually end up in a different spectrum of disorders.

The search, which was completed in May 2024, was limited to English- or Spanish-language journal articles involving in vitro, human, and/or animal subjects, with no time filter applied. Rayyan software v. 1.5 (Qatar Computing Research Institute, HBKU, Doha, Qatar) [41] was used to expedite the initial screening of abstracts and titles using a semi-automated process while maintaining a high level of usability. This web-based tool was used to facilitate efficient screening, selection, and collaboration on research articles through filtering and tagging.

Inclusion criteria were scientific articles in English or Spanish that addressed mitochondrial OXPHOS in the field of SCZ. Studies using exogenous components to modulate OXPHOS in their study models were excluded, as the focus of this review is solely on the pure etiopathogenesis of the disease, without the presence of potential external artifact factors. Therefore, studies on neurodegeneration in general, neuropsychiatric disorders other than SCZ (bipolar disorder, attention-deficit/hyperactivity disorder, among others), neuronal signaling, protein pathways or proteomics (in general), or immune pathways, proteomics and metabolomics in psychiatry (in general), mitochondrial dysfunction in mental/psychiatric disorders (in general), AKT/GSK3 pathways, monoamine oxidase and phosphatidylserine protein levels, multiple sclerosis therapy, oxidative stress (without OXPHOS assessment), mitochondrial dynamics, expression of mitochondrial-related genes (unless directly related to OXPHOS components), clozapine or antipsychotics as the causal factors of observed OXPHOS alterations, Alzheimer’s disease, Toxoplasma gondii infection, cardiovascular diseases, traumatic brain injury, and exogenous OXPHOS (succinate or cytochrome c, among others) and redox stress inducers (paraquat or hydrogen peroxide, among others) have been discarded in the present review.

Although this narrative review includes articles discussing oxidative stress or oxidative damage due to their close relationship with OXPHOS deficits, the studies listed in the outcomes focus exclusively on those that strictly analyze the OXPHOS System (CI to CIV and ATPase), as this represents the primary target of interest in this study. However, this does not preclude references to oxidative stress-related data in certain parts of the introduction or result interpretation where relevant. The same applies to other aspects such as mitochondrial density or mitochondrial metabolism. This is because cellular bioenergetics—neither glycolysis nor other mitochondrial functions—are mechanistically and functionally inseparable from OXPHOS, although OXPHOS is the specific focus of this review, as observed in the compilation of studies presented in the following section.

## 3. Key Findings and Insights

The main distinguishing feature of this study is the focus on OXPHOS outcomes observed in SCZ models, excluding any other mitochondrial parameters beyond the OXPHOS pathway (such as oxidative damage, oxidative stress, or mtDNA alterations, among others) unless they are accompanied by impairment at the level of OXPHOS. Molecular parameters such as oxygen consumption or ATP synthesis or levels are considered part of OXPHOS, since the former is performed at the level of CIV and the latter is generated at the level of CV or ATPase [1].

When, in the reviewed studies, alterations at the level of OXPHOS triggered or were closely associated with other molecular abnormalities, these alterations were not excluded from the remarks (provided that such findings were always accompanied by a description of OXPHOS damage). For instance, mtDNA variations or oxidative stress variables were included in the results [42], as mtDNA variants may lead to OXPHOS dysfunction, and OXPHOS dysfunction may promote oxidative stress, respectively. Homologously, apoptosis has also been mentioned here [43], as it is part of the downstream effects of OXPHOS dysfunction. Other examples include homocysteine, lactate levels, and acetyl-CoA levels, which have a direct metabolic impact on OXPHOS: (i) iron-sulfide proteins obtain sulfide from L-cysteine (which is dependent on homocysteine levels). Since OXPHOS contains diverse iron-sulfide proteins, low levels of L-cysteine may lead to suboptimal iron-sulfide cluster formation and subsequent deficits in OXPHOS in the SCZ [44]; (ii) lactate levels may increase if pyruvate is not properly internalized into the mitochondria due to mitochondrial dysfunction (such as OXPHOS deficits) [1]; (iii) acetyl CoA serves as a promoter of the Krebs cycle, which generates reducing power products that serve as feeders for OXPHOS [1].

The main findings of this review are summarized in Table 1. Studies are listed chronologically divided as reviews or original data. Although most of the reviews include different study models, those focusing only on human subjects have been indicated as R(H). Studies with original data have been clustered according to the study model, including human (H), in vitro, or in vivo studies using mouse or rat SCZ models. Interestingly, OXPHOS deficits were found across species. Complex I deficiencies, considered the gold standard for many neurodegenerative processes such as Parkinson’s disease and SCZ, have been described in humans [45], including both peripheral and postmortem brain samples [46], as well as observed in the rat model [45]. The animal models allow for approaches unthinkable at the human level. Social isolation in rats led to SCZ-like behaviors, providing an early perspective on the disease by focusing on a negative symptom. In this model, increased lactate production by astrocytes clearly indicated a mitochondrial dysfunction compatible, with OXPHOS impairment [47]. Neuroblastoma cell cultures also serve as a useful platform to model SCZ. On the one hand, they allow easy testing of exogenous compound toxicity. The fact that dopamine toxicity led to mitochondrial complex I inhibition [48] serves as evidence that the dopaminergic etiopathogenesis of SCZ may be closely related to OXPHOS dysfunction. Genetically modified neuroblastoma using knockdown SHSY5Y cells has also been used to study SCZ and confirmed OXPHOS dysfunction with impaired oxygen consumption and ATP synthesis [49].

### 3.1. Postmortem Evidence of OXPHOS Alterations in Brains from Individuals with SCZ (mRNA, Proteins, Metabolites)

Human research shows deficits in OXPHOS in SCZ from imaging, transcriptomic, proteomic, and metabolomic studies. In postmortem brains of SCZ individuals, there is a decrease in cytochrome c oxidase activity in the nucleus caudatus and a reduction in the cortex gyrus frontalis compared to controls [44,65]. There is a significant decrease in the complex I activity in peripheral blood mononuclear cells of individuals with SCZ compared to controls [44,66]. There is a downregulation of nuclear mRNA molecules and proteins involved in OXPHOS and a decrease in high-energy phosphates in SCZ brains [4,44]. In postmortem SCZ brains of individuals, complex IV was significantly reduced in the frontal cortex and temporal cortex, and complex I + III activity was significantly reduced in the temporal cortex and basal ganglia [44,67]. Deficits in OXPHOS would reduce ATP levels because the OXPHOS pathway synthesizes ATP. There are decreases in ATP in the frontal lobes of mainly naive individuals with SCZ compared to controls [44,68].

Understanding the relationship between mitochondrial OXPHOS and SCZ serves three main purposes: (I) to improve our understanding of the aetiopathogenesis of SCZ, (II) to improve current therapeutic approaches towards more specific targets closely related to the aetiopathogenesis of the disease, and (III) to improve the clinical diagnosis of the disease. In this context, first, due to the apoptotic features observed in SCZ [15], it is conceivable that OXPHOS mitochondrial alterations could be a core pathophysiological etiopathogenic cause of the disease. Second, the mapping of OXPHOS alterations around the SCZ could be a first step for further improved mitochondrially targeted therapeutic or mitotherapeutic approaches [39]. Third, a comprehensive blood analysis could improve the diagnosis of patients with SCZ [61]. Indeed, differences in plasma metabolites between 22qDS children and controls reflect a shift from OXPHOS to glycolysis (higher lactate/pyruvate ratio), accompanied by an increase in reductive carboxylation of α-ketoglutarate (increased concentrations of 2-hydroxyglutate, cholesterol, and fatty acids) [9]. Neurometabolites such as N-acetylaspartate (NAA), creatine (Cr), and choline (Cho) are closely linked to mitochondrial OXPHOS. NAA is synthesized within mitochondria and reflects ATP production, while Cr is involved in energy transfer. Reduced NAA levels in SCZ suggest mitochondrial dysfunction and impaired OXPHOS, leading to bioenergetic deficits. Altered Cr levels may indicate disruptions in cellular energy homeostasis, whereas stable Cho levels suggest preserved membrane integrity. These findings support the hypothesis of mitochondrial impairment and a metabolic shift from OXPHOS to glycolysis in SCZ. A systematic review and meta-analysis assess neurometabolite alterations in SCZ and bipolar disorder using proton magnetic resonance spectroscopy (^1^H-MRS), focusing on NAA, Cho, and Cr levels across brain regions. The study found significant reductions in NAA levels in the thalamus and frontal lobe in SCZ and in the basal ganglia in bipolar disorder. Cr and Cho levels remained unchanged in both disorders. These findings suggest potential neurometabolic abnormalities, emphasizing the need for larger, well-controlled studies [69].

### 3.2. Central and Peripheral Systems

Indices of endogenous oxidative and antioxidative processes measured in the plasma of SCZ patients suggest a possible role for increased oxidative stress and decreased enzymatic antioxidants, as potential alterations in the core pathophysiology of SCZ. Beyond its role in energy metabolism, OXPHOS dysfunction can mechanistically disrupt neural circuits by impairing synaptic transmission, altering neurotransmitter release, and diminishing neuronal excitability. Defective ATP production compromises the maintenance of ion gradients essential for action potentials, while increased oxidative stress and calcium dysregulation further exacerbate synaptic dysfunction and network instability. These alterations may contribute to the deficits in connectivity and synchronization observed in SCZ.

Alterations in the function of OXPHOS measured in peripheral tissues, such as blood (platelets, lymphocytes), or muscle [54,66], could reflect similar dysfunctions occurring in the brain of patients with SCZ.

The underlying reason is similar: a fundamental metabolic or bioenergetic dysfunction can have systemic manifestations. If mitochondria in the brain of individuals with SCZ exhibit deficiencies in OXPHOS, these same deficiencies could be present, although perhaps to a lesser extent, in other peripheral tissues where mitochondria also play a crucial role in energy production. For example, inhibition of complex I has been reported in platelets of SCZ patients medicated with antipsychotics [54], suggesting a peripheral effect of centrally acting drugs that have also been observed to have effects on cerebral OXPHOS.

Therefore, measurements of the activity of OXPHOS complexes or related markers in peripheral samples could serve as complementary tools to investigate central mitochondrial dysfunction in SCZ and potentially aid in diagnosis or in monitoring treatment response. However, it is crucial to consider that peripheral results can be influenced by various factors, and the direct correlation with specific brain alterations still requires further investigation.

### 3.3. Alterations in Several Complexes

In the context of SCZ, the evidence for alterations of all complexes (I to V) is not conclusive; however, it is evident that alterations exist in several of them. Significantly reduced activity in complexes I and IV in neurons derived from patients with 22q11DS and SCZ were found, although no changes in activity in complexes II, III, and V were recorded [22]. Alterations in specific complexes have been described throughout the literature. Decreases in the activity of complex I and IV and alterations in gene expression have been reported [45]. Interestingly, gene expression studies suggest downregulation of components of complexes I, III, IV, and V [57,58]. In general, there is evidence of dysfunction, mainly in complexes I and IV, in SCZ, and some indications of possible alterations in the other complexes at a genetic level, but not a consistent confirmation of functional dysfunction in all of the complexes. Recent studies have shown that individual respiratory complexes can assemble into higher-order structures known as supercomplexes, which enhance electron transfer efficiency and stabilize the mitochondrial membrane. The proper formation of respiratory supercomplexes relies on the structural integrity of mitochondrial cristae, which are organized by the mitochondrial contact site and cristae organizing system (MICOS) complex. Disruption of MICOS components, such as DISC1—a protein implicated in SCZ—may impair cristae architecture and consequently compromise oxidative phosphorylation efficiency [49].

### 3.4. Energy Metabolism

The metabolic shift from less oxidative (OXPHOS-based) to more glycolytic processes for energy production, has been observed in SCZ research models (including both human studies and animal models) and in many other diseases [9,23]. In such studies, the disease model carries a different genetic burden that is ultimately reflected in changes at the mitochondrial functional level of OXPHOS. In the brain of mice with a 3q29Del background, a selective decrease in the components of complexes II and IV was observed, indicating a shift in the stoichiometry of the respiratory chain complexes [23], while in the plasma of children with 22q11.2DS, different mitochondrial metabolites (glucose, cholesterol, proline, lactate/pyruvate ratio) were found [9]. With regard to the relevance of the metabolic state, in which mitochondria play a pivotal role as an influencing factor in the development of the disease, it is crucial to consider the intricate interplay between mitochondrial function and cellular metabolism. Dysfunctional mitochondria can lead to disruptions in cellular energetics, oxidative stress, and altered signaling pathways, all of which have been implicated in the pathogenesis of SCZ, and literature reports that impaired brain energy metabolism (along with developmental abnormalities, abnormal neurotransmission, and neuronal connectivity) may account for the pattern of multifactorial inheritance in SCZ [50].

Understanding how mitochondrial function affects metabolic pathways and contributes to disease development is essential for identifying novel therapeutic targets and developing effective interventions for mitochondrial-related disorders.

### 3.5. Other Molecules Related to OXPHOS

Direct upstream precursors of OXPHOS may also play a key role in the pathogenesis of SCZ. Homocysteine has been implicated in mitochondrial dysfunction by inducing oxidative stress and cellular apoptosis. It has also been suggested to interfere with mitochondrial energy production, contributing to mitochondrial dysfunction and its associated adverse health effects. The severity of SCZ has been positively correlated with homocysteine levels [44,70] due to low activity of the transsulfuration pathway that metabolizes homocysteine in the synthesis of L-cysteine, an amino acid that plays an important role in OXPHOS by serving as a precursor for glutathione synthesis. Another example of an affected OXPHOS precursor is lactate. Briefly, lactate is converted to pyruvate, and pyruvate is further converted to acetyl-CoA in the mitochondria. Acetyl-CoA enters the Krebs cycle, which produces molecules such as NADH and FADH2 that are essential for OXPHOS function. In a rat model of social isolation that examines the balance of glucose metabolism, rats in the isolated housing group showed impaired prepulse inhibition and increased lactate levels in the prefrontal cortex [47]. In a clinical study, patients with SCZ had higher lactate elevations (a condition related to suboptimal mitochondrial function) than healthy subjects during exercise, suggesting that lactate metabolism may be a target for potential therapeutic treatments [71].

### 3.6. Pharmacological Considerations

The present review has excluded SCZ studies in vitro or in animal models based on the addition of exogenous OXPHOS modulators to avoid the possible influence of this external factor on the observed biological and mitochondrial processes. Pharmacological exogenous compounds specifically modulating OXPHOS were excluded due to obvious potential interference with the observed results, but this is not the case for antipsychotic approaches.

Although this review is mainly focused on the etiopathogenesis of SCZ, the fact that antipsychotic drugs may target etiopathogenic pathways entails the exploration of whether these drugs affect OXPHOS pathways as a possible strategy of interest in this topic.

The actions of antipsychotic drugs may provide a clue to understanding the neuropathology of SCZ, as described in a seminal study [72], also from a mitochondrial and OXPHOS perspective. Indeed, differential mitochondrial protein expression in the cortex and hippocampus of animal models has been associated with exposure to antipsychotic treatment. Differential mitochondrial protein expression was assessed by two-dimensional (2D) gel electrophoresis for four groups of a mouse model exposed to chlorpromazine, clozapine, quetiapine, and a control group. A total of fourteen proteins, six of which belong to the respiratory ETC of OXPHOS, showed significant changes in abundance, including NADH dehydrogenase (ubiquinone) 1 alpha subcomplex 10 (Ndufa10), NADH dehydrogenase (ubiquinone) flavoprotein 2 (Ndufv2), NADH dehydrogenase (ubiquinone) Fe-S protein 3 (Ndufs3), F1-ATPase beta subunit (Atp5b), ATPase, H^+^ transporting, lysosomal, beta 56/58 kDa, isoform 2 (Atp6v1b2), and ATPase, V1 subunit A, isoform 1 (Atp6v1a1) [73]. These data clearly suggest a mitochondrial involvement in the biological pathways underlying SCZ. The treatment-induced changes at the OXPHOS level may indicate the suitability of using samples from treatment-naïve patients to study the etiopathogenesis and experimental pharmacology of the disease; moreover, they are also an indicator that these pathways are intimately involved in the disease. This is because antipsychotic treatment could potentially interfere precisely with the very same biological processes that underlie the disease.

Emerging single-cell technologies have revealed that mitochondrial dysfunction in SCZ may exhibit cell-type specificity, affecting different neuronal and glial populations in distinct ways. Such findings highlight the importance of considering mitochondrial alterations not as uniform across the brain, but rather as context-dependent phenomena that may vary according to cellular identity, metabolic demands, and regional vulnerabilities. Incorporating this perspective will be crucial for developing more precise therapeutic strategies in the future.

### 3.7. Common OXPHOS Alterations in SCZ and Other Brain Disorders

Research suggests that mitochondrial dysfunction, including alterations in the OXPHOS system, is not unique to SCZ and may represent a shared vulnerability across several psychiatric and neurodegenerative conditions [74]. For instance, studies have indicated mitochondrial involvement in bipolar disorder [24], and specific deficits in ATP synthase and cytochrome c oxidase have been observed in Alzheimer’s disease [54]. Furthermore, a module of genes enriched for mitochondrial function has shown downregulation across autism spectrum disorder (ASD), SCZ, and bipolar disorder, potentially linking energetic balance to these conditions 4. In line with this, iPSC-derived neurons from individuals with 22q11.2 deletion syndrome, a genetic risk factor for SCZ, also exhibit mitochondrial deficits including reduced activity in oxidative phosphorylation complexes [22]. These convergent findings underscore the possibility of shared mitochondrial and OXPHOS impairments as a common biological underpinning in the pathophysiology of several brain disorders.

### 3.8. Mitochondrial Genome Alterations

Despite the fact that this study only focuses on compiling results (Table 1) from studies explicitly describing OXPHOS deficits, it is worth noting that not all studies have exclusively analyzed the enzymatic functions of the constituent complexes of OXPHOS and alterations at other mitochondrial levels are directly related to this dysfunction. MtDNA deletions have recently been identified in the postmortem tissue of patients with SCZ [75]. The description of deletions in the mitochondrial genome [42] or the depiction of oxidative stress illustrates this point, since on the one hand it is known that a low level of mtDNA copies can lead to defects in the function of the ETC and OXPHOS, and on the other hand, dysfunction of OXPHOS can promote and correlate with oxidative damage in cells.

### 3.9. Future Directions

Future research should prioritize the integration of single-cell omics approaches to dissect the cell-type specific features of mitochondrial dysfunction in SCZ, and primary models derived from patients, including neuronal derived from stem cells. Elucidating the precise mechanisms linking OXPHOS abnormalities to neural circuit disorganization remains a critical challenge. Here, functional studies investigating synchrony and connectivity in neuronal circuits, for example through calcium image and microelectrode techniques between different neuronal types, may be particularly helpful. Furthermore, the development of targeted therapies aimed at restoring mitochondrial function, enhancing cristae architecture, or stabilizing respiratory supercomplexes holds promise. Longitudinal studies investigating mitochondrial resilience and vulnerability across disease stages are also essential to design timely interventions.

## 4. Concluding Remarks

The elucidation of the mitochondrial implication, specifically of OXPHOS, in the etiopathogenesis of SCZ, analogous to many other pathological processes, remains enigmatic in terms of defining its causal or consequential involvement. Whether these mitochondrial dysfunctions are the cause or the consequence of the disease is still uncertain—a classic chicken-and-egg dilemma. Further studies in early stages or in at-risk populations would be very useful to clarify this. However, as reviewed, it has been widely described that these processes are significantly altered in the disease. Despite the challenge of drawing a definitive conclusion, the potential impact of these mitochondrial processes underscores their importance in understanding and potentially targeting therapeutic interventions for SCZ and related disorders.

## 5. Highlights

Compiled findings indicate the following: (i) OXPHOS dysfunction has been confirmed in SCZ pathology by various biological systems at the molecular level of RNA, protein, metabolites, etc.; (ii) OXPHOS dysfunction has been described in both central and peripheral systems and organs (including the brain as the main target organ of the disease); (iii) OXPHOS dysfunction is present in several complexes that make up the ETC and ATP synthase (from complex I to complex V); (iv) OXPHOS system alterations represent common and extrapolatable findings across different psychiatric and neurodegenerative disorders.

## Figures and Tables

**Table 1:** Summary of the main outcomes found in the studies selected for this investigation.

Title	Reference	Aim of the Study	Oucomes and Remarks	Type/Model
Mitochondrial dysfunction in schizophrenia: a possible linkage to dopamine	[50]	To review several independent lines of evidence suggesting the involvement of mitochondrial dysfunction in SCZ	Provides evidence that impaired brain energy metabolism, developmental abnormalities, abnormal neurotransmission, and neuronal connectivity may account for the pattern of multifactorial inheritance in SCZ	H (brain)
Mitochondria, synaptic plasticity, and schizophrenia	[51]	To propose that mitochondrial dysfunction in SCZ may cause, or result from, abnormalities in the processes of plasticity in this disorder	Data from biochemical and genetic analysis of SCZ patients showed mitochondrial dysfunction, including mitochondrial hypoplasia, OXPHOS alterations, and altered mitochondrial-related gene expression.	H (brain)
Psychiatric disorder biochemical pathways unraveled by human brain proteomics	[52]	To collect and analyze data on differentially expressed proteins found in postmortem brain studies of SCZ, bipolar disorder, and major depressive disorder	Different psychiatric disorders including bipolar, depression, and SCZ share about 21% of proteins affected, and though most are related to energy metabolism pathways deregulation and mitochondrial function (OXPHOS mainly found in depression).	H (brain)
Schizophrenia: a disorder of broken brain bioenergetics	[53]	To explore bioenergetic dysfunction in SCZ, summarizing evidence on metabolic impairments in insulin signaling, glycolysis, the pentose-phosphate pathway, the TCA cycle, and OXPHOS	Reduced CI and CIV activity and decreased mitochondrial gene expression in the dorsolateral prefrontal cortex. Deficits in CI activity and altered ATP production in the anterior cingulate cortex; reduced CIV activity and decreased bioenergetic efficiency in the hippocampus. Reduced CI activity and altered redox homeostasis in the *striatum* (*caudate nucleus* and *putamen*). Downregulation of OXPHOS-related mitochondrial genes in the thalamus.	H (brain)
Mitochondrial complex I subunits expression is altered in schizophrenia: a postmortem study	[46]	To analyze three subunits of mitochondrial CI at the mRNA and protein levels in postmortem brain samples from the prefrontal and the ventral parietooccipital cortex of patients with SCZ, major depression, bipolar disorder, and control subjects	Both mRNA and protein levels of the 24-kDa and 51-kDa subunits of CI were significantly decreased in the PFC, but increased in the ventral parietooccipital cortices of SCZ patients. In the latter region, protein levels of both subunits were also increased in bipolar patients as well, consistent with the significant overlap in clinical symptoms between SCZ and bipolar patients.	H (brain)
Multivariate meta-analyses of mitochondrial complex I and IV in major depressive disorder, bipolar disorder, schizophrenia, Alzheimer’s disease, and Parkinson’s disease	[54]	To evaluate mitochondrial CI and CIV dysfunction across major psychiatric disorders (SCZ, bipolar disorder, major depressive disorder) and neurodegenerative diseases (Alzheimer’s and Parkinson’s). The study aims to identify shared and distinct patterns of mitochondrial impairment.	CI: reduced subunit expression in the striatum and frontal cortex, suggesting mitochondrial dysfunction in these regions. CIV: decreased enzymatic activity in the frontal cortex, but increased activity in the basal ganglia (nucleus accumbens, globus pallidus, and putamen). Conclusion in SZ: findings indicate region-specific and heterogeneous alterations in CI and CIV.	H (brain)
Postmortem studies on mitochondria in schizophrenia	[55]	To examine mitochondrial abnormalities in postmortem brains of individuals with SCZ, focusing on ultrastructural changes, enzyme activity, and regional differences	Reduced mitochondrial density in the anterior cingulate cortex and striatum, with region-specific alterations in OXPHOS complexes. Findings suggest mitochondrial dysfunction varies by brain region, cell type, treatment response, and symptom profile, reinforcing its role in SCZ pathophysiology.	H (brain)
Shared molecular neuropathology across major psychiatric disorders parallels polygenic overlap	[56]	To investigate shared and distinct gene expression patterns across major psychiatric disorders (SCZ, bipolar disorder, autism, depression, and alcoholism) using transcriptomic profiling of postmortem brain tissue	Overlapping transcriptional dysregulation, particularly in synaptic and mitochondrial-related genes, across SCZ, bipolar disorder, and autism.	H (brain)
Distinctive transcriptome alterations of prefrontal pyramidal neurons in schizophrenia and schizoaffective disorder	[57]	To investigate cell type-specific gene expression in pyramidal neurons from the dorsolateral PFC in SCZ and schizoaffective disorder, focusing on mitochondrial and ubiquitin–proteasome system pathways	Deficits in mitochondrial gene expression were observed in pyramidal neurons of the dorsolateral prefrontal cortex in SCZ. These alterations were more prominent in layer 3, where reductions in the expression of key OXPHOS system genes were found. Deficits in the expression of CI genes and other ATP production-related genes suggest a hypometabolic state in pyramidal neurons of this region.	H (brain)
Transcriptome alterations of prefrontal cortical parvalbumin neurons in schizophrenia	[58]	To examine transcriptomic alterations in parvalbumin interneurons from the dorsolateral PFC in SCZ using laser microdissection and microarray analysis	Mitochondrial dysfunction and OXPHOS deficits in parvalbumin neurons, with >85% of OXPHOS-related genes showing reduced expression. These alterations, distinct from pyramidal neurons, suggest cell-type-specific bioenergetic impairments contributing to SCZ pathology.	H (brain)
Mitochondrial dysfunction in schizophrenia: evidence for compromised brain metabolism and oxidative stress	[59]	To investigate mitochondrial dysfunction and oxidative stress in postmortem PFC tissue from individuals with SCZ	Significant downregulation of OxPhos-related genes and proteins, particularly in CI, CIII, and CIV, alongside elevated oxidative stress markers. These alterations suggest impaired energy metabolism and increased oxidative stress as key contributors to SCZ pathophysiology.	H (brain)
The interplay between mitochondrial complex I, dopamine, and Sp1 in schizophrenia	[45]	To review the evidence supporting mitochondrial dysfunction in SCZ	The study provides evidence of abnormalities in mitochondrial CI, which plays an important role in controlling OXPHOS activity.	H (brain and blood)
Mitochondrial citrate transporter-dependent metabolic signature in the 22q11.2 deletion syndrome	[9]	To investigate whether mitochondrial outcomes and metabolites of 22qDS children segregate with the altered dosage of one or more of these mitochondrial genes that contribute to 22qDS etiology and/or morbidity	Metabolite differences between 22qDS children and controls reflected a shift from oxidative phosphorylation to glycolysis (higher lactate/pyruvate ratio) accompanied by an increase in reductive carboxylation of -ketoglutarate (increased levels of 2-hydroxyglutaric acid, cholesterol, and fatty acids). Altered metabolism in 22qDS reflected a critical role in the haploinsufficiency of the mitochondrial citrate transporter *SLC25A1*, which was further enhanced by HIF-1, MYC, and metabolite controls.	H (blood, PBMC)
Disrupted in schizophrenia 1 (DISC1) is a constituent of the mammalian mitochondrial contact site and cristae organizing system (MICOS) complex, and is essential for oxidative phosphorylation	[49]	To elucidate the role of DISC1 in OXPHOS function in DISC1 knockdown SHSY5Y nb cells	OXPHOS complexes and supercomplexes are partially disassembled in nb cells that exhibit impaired oxygen consumption, ATP synthesis, and mitochondrial membrane potential. Transfection of recombinant full-length human DISC1 restores MICOS complex assembly and rescues OXPHOS function.	Hc (nb) and M
iPSC-derived homogeneous populations of developing schizophrenia cortical interneurons have compromised mitochondrial function	[60]	To generate homogeneous populations of developing cortical interneurons from human SCZ and control iPSC lines	SCZ cortical interneurons, but not SCZ glutamatergic neurons, show dysregulated OXPHOS-related gene expression accompanied by impaired mitochondrial function.	H (iPSC-n)
Mitochondrial deficits in human iPSC-derived neurons from patients with 22q11.2 deletion syndrome and schizophrenia	[22]	To investigate mitochondrial dysfunction in neurons derived from iPSCs from patients with 22q11.2 deletion syndrome and SCZ. The authors examine ATP production, oxidative phosphorylation complex activity, and mitochondrial protein expression to explore their role in neuronal dysfunction	Significant reductions in ATP levels and oxidative phosphorylation CI and IV activity in patient-derived neurons. These deficits were linked to decreased mitochondrial-encoded protein expression, partially attributed to *MRPL40* haploinsufficiency.	H (iPSC-n)
The correlation-base-selection algorithm for diagnostic schizophrenia based on blood-based gene expression signatures	[61]	To improve clinical diagnosis of SCZ by collecting whole blood gene expression data	The study reports that the analysis of gene expression in whole blood by their proposed model could be a useful tool for diagnosing SCZ. Samples were divided into 10 groups, and cross-validation showed that the model we constructed achieved nearly 100% classification accuracy. Characterized genes enriched in OXPHOS, among others, were identified.	H (blood)
Expression of actin- and oxidative phosphorylation-related transcripts across the cortical visuospatial working memory network in unaffected comparison and schizophrenia subjects	[62]	To understand the relationship between the dorsolateral prefrontal cortex, altered expression of transcripts for actin assembly, and mitochondrial OXPHOS in the context of visuospatial working memory in SCZ patients	All OXPHOS transcripts showed −15 to −22% lower levels in RNAseq from SCZ subjects.	H
Mitochondrial complex I subunits are altered in rats with neonatal ventral hippocampal damage but not in rats exposed to oxygen restriction at neonatal age	[63]	To decipher whether mitochondrial CI abnormalities in SCZ are a core pathophysiological process or drug-induced	Hippocampal lesion induced a significant prepubertal increase and postpubertal decrease in all three subunits of CI as compared to sham-treated rats, whereas no change was observed in the cingulate cortex. Neonatal exposure to hypoxia did not affect protein levels of any of the three subunits in the PFC.	Rat
Juvenile social isolation leads to schizophrenia-like behaviors via excess lactate production by astrocytes	[47]	To further explore the balance of glucose metabolism based on previous transcriptomic analysis in a rat model of social isolation	Compared to the social rearing group, rats in the isolated rearing group showed impaired prepulse inhibition and increased lactate levels in the PFC.	Rat
Energization by multiple substrates and calcium challenge reveal dysfunctions in brain mitochondria in a model related to acute psychosis	[37]	To analyze mitochondrial function under conditions of isolated or multiple respiratory substrates using brain mitochondria isolated from MK-801-exposed mice	Mitochondria may compensate for deficiencies in a single mitochondrial complex when oxidizing multiple substrates simultaneously; Ca^2+^ handling is compromised in MK-801-exposed mice, resulting in a loss of phosphorylative capacity and an increase in H_2_O_2_ production.	M
Cross-species analysis identifies mitochondrial dysregulation as a functional consequence of the schizophrenia-associated 3q29 deletion	[23]	To profile the transcriptomes of isogenic cortical organoids from the 3q29Del aged for 2 and 12 months, as well as perinatal mouse isocortex, all at single-cell resolution	Systematic pathway analysis suggested dysregulation of mitochondrial function and energy metabolism. Lack of metabolic flexibility and a contribution of the 3q29 gene PAK2.	M
Fisetin, potential flavonoid with multifarious targets for treating neurological disorders: an updated review	[64]	To evaluate the potential mechanisms and pharmacological effects of fisetin in the treatment of several neurological diseases, including SCZ	Fisetin promotes the elevation of Acetyl CoA and has been reported to improve cognitive function by promoting synaptic plasticity.	Rat

ATP, adenosine triphosphate; Acetyl CoA; acetyl coenzyme A; CI, complex I; CI+III, complex I+III; CIV, complex IV; *DISC1*, Disrupted in schizophrenia 1; H, human subject; Hc, human cell; iPSC, induced pluripotent stem cell; iPSC-n, induced pluripotent stem cell-derived neuron; M, mouse; MICOS, mitochondrial contact site and cristae organizing system; MtDNA, mitochondrial DNA; nb, neuroblastoma SHSY5Y cell; OXPHOS, oxidative phosphorylation system; PBMC, peripheral blood mononuclear cell; PFC, prefrontal cortex; PRS, polygenic risk score; SCZ, schizophrenia; R, review; TCA, tricarboxylic acid cycle. The studies in this table are organized based on study type or model (e.g., reviews, human tissue studies, animal models (indicated in the rightmost column)). Only studies reporting deficits in OXPHOS enzyme complexes are included in this table. Findings on neurometabolites and other related markers are detailed in the discussion section.

## Data Availability

No new data were created or analyzed in this study. Data sharing is not applicable to this article.

## Abbreviations

The following abbreviations are used in this manuscript:

ADP	Adenosine diphosphate
ASD	Autism spectrum disorder
ATP	Adenosine triphosphate
Cho	Choline
CNV	Copy number variants
CoQ	Coenzyme Q
Cr	Creatine
CI	Complex I or NADH dehydrogenase
CII	Complex II or succinate dehydrogenase
CIII	Complex III or cytochrome reductase
CIV	Complex IV or cytochrome c oxidase
CV	Complex V or ATP synthase
ETC	Electron transport chain
GWAS	Genome-wide association studies
iPSC	Induced pluripotent stem cells
MRS	Magnetic resonance spectroscopy
MtDNA	Mitochondrial DNA
NAA	N-acetylaspartate
NAD+	Nicotinamide adenine dinucleotide
NADH	Reduced form of nicotinamide adenine dinucleotide
OXPHOSI	Oxidative phosphorylation system
PBMC	Peripheral blood mononuclear cells
RCV	Rare coding variants
ROS	Reactive oxygen species
SCZ	Schizophrenia
